# The protective effect of taurine, piracetam and vinpocetine on etoposide-induced inflammation and brain injury in the serum of female albino rats

**DOI:** 10.3332/ecancer.2023.1499

**Published:** 2023-01-23

**Authors:** Arwa Salam Mohammed, Ansam N Al-Hassani, Rafal Abdulrazaq Alrawi, Rawaz D Tawfeeq

**Affiliations:** 1 Department of Pharmacology and Toxicology, College of Pharmacy, Hawler Medical University, Erbil 44001, Iraq; 2 Department of Clinical Analysis, College of Pharmacy, Hawler Medical University, Erbil 44001, Iraq

**Keywords:** etoposide, toxicology, immunohistochemistry, brain, glial fibrillary acidic protein

## Abstract

Etoposide (ETP) is one of the leading antitumour agents in cancer chemotherapy. Many studies have reported on ETP-induced peripheral neuropathy; however, few reports have focused on its brain toxicity. The current research investigates the protective potential of taurine, piracetam and vinpocetine on serum biomarkers associated with inflammation and brain injury induced by ETP in a rodent model. A total of 30 female albino rats were equally divided into five groups; the 1^st^ and 2^nd^ groups were the control and ETP-treated groups, respectively, while the 3^rd^, 4^th^ and 5^th^ groups were ETP-treated rats cotreated with taurine, piracetam and vinpocetine, respectively. Administration of ETP reduced body weight significantly, enhanced production of serum proinflammatory cytokines including tumour necrosis factor-alpha, interleukin-1 beta (IL-1β) and IL-6 and decreased glutathione serum levels. Moreover, ETP treatment resulted in upregulation of glial fibrillary acidic protein expression and histopathological alterations in the rats’ brain compared to the control group. Co-treatment with taurine, piracetam and vinpocetine counteracted ETP-induced brain injury and altered serum biomarkers levels. We concluded that co-treatment with vinpocetine could serve as a complementary therapeutic agent in reducing brain injury and toxicity induced by ETP.

## Introduction

Despite advances in developing new anticancer drugs, and a continuous increase in the number of cancer survivors, the off-target toxicity of anticancer drugs that eventually affects the quality of life is the main challenge facing oncologists [1–3]. Chemotherapeutic drugs are cytotoxic agents that stop the abnormal growth of cancerous cells. However, these drugs cannot differentiate between cancer cells and normal cells leading to off-target toxicities. Therefore, new therapies are constantly being developed and searched for in order to minimise the life-threatening side effects of the current anticancer drugs without affecting their efficacies [4–7].

Etoposide (ETP) is a semisynthetic derivative of podophyllotoxin and one of the leading antitumour agents in cancer chemotherapy. It is used alone or in conjugation with other antineoplastic drugs against a wide variety of malignancies [8–10]. Yet, the clinical use of ETP is often limited due to its toxic effects on organs including liver, kidney, heart and lungs [11–16]. ETP exerts its cytotoxic effect by impairing the activity of topoisomerase II enzyme which eventually results in single- and double-stranded DNA breaks during DNA replication. This impairment of DNA replication activates a cascade of events that eventually leads to cell death via apoptosis [10, 17, 18]. In addition to that, the quinone metabolite of ETP has enhanced antitumour activity and oxidising ability than its parent drug [10, 17].

Although ETP poorly penetrates the Blood-Brain Barrier (BBB) [19, 20], animal studies have shown that intracarotid infusion of ETP is capable of disrupting the BBB and inducing acute neurotoxicity in high doses [21–23]. Furthermore, a human study which investigated the incidence of peripheral neuropathy in patients receiving ETP concluded that peripheral neurotoxicity of moderate severity is a possible complication of high-dose ETP [24]. Patients receiving multi-drug chemotherapy containing ETP can develop chemo brain and neurocognitive dysfunction [25, 26]. The mechanism underlying ETP-induced neurotoxicity remains to be fully explored, but previous studies on doxorubicin-induced neurotoxicity point to redox active quinone metabolite as one of the major contributing factors [27, 28]. Furthermore, it is suggested that it could be through alteration of the oxidative/antioxidative status and enhanced production of peripheral proinflammatory cytokines which can cross the BBB and cause neuroinflammation [1]. Therefore, neuroprotective agents that increase the activity of endogenous antioxidant enzymes and decrease proinflammatory cytokines may be promising against ETP-induced neurotoxicity [29, 30]. This study considered the use of taurine, piracetam and vinpocetine to mitigate ETP-induced toxicity in the brain of rats.

Taurine (2-aminoethanesulfonic acid) is a semi-essential amino acid synthesised in the human body and found abundantly in the nervous system and excitable tissues; it has a crucial function in neuronal cell development. Taurine has a therapeutic role in several neurological disorders including; depression, anxiety, stroke, epilepsy, traumatic brain injury and neurodegenerative diseases. The neuroprotective effect of taurine is attributed to its multitude of actions, which includes antioxidation, endoplasmic reticulum stress inhibition, glutamate excitotoxicity inhibition, calcium overload reduction, agonising gamma-aminobutyric acid (GABA), glycine and N-methyl-D-aspartate (NMDA) receptors, anti-apoptotic, anti-inflammatory and osmoregulatory activities [31–34]. Delivery of exogenous taurine to the brain is limited by the BBB. However, during brain injury, the permeability of the BBB increases. Therefore, administered taurine can freely cross the BBB and reach injured neurons and astrocytes via the taurine transporter to exert its neuroprotective effect [32, 35].

The nootropic agent, piracetam (2-oxo-pyrrolidone) is a cyclic derivative of GABA neurotransmitter that is widely used in treating cerebrovascular and neurodegenerative diseases. Studies have shown that piracetam decreases neuronal damage and exhibits neuroprotective effects by reducing oxidative stress and neuroinflammation. The exact mechanism of action by which it achieves this is not fully understood. However, it has been documented that piracetam is an allosteric modulator of α-amino-3-hydroxy-5-methyl-4-isoxazolepropionic acid and an NMDA excitatory neurotransmitter that facilitates the function of acetylcholine neurotransmitter which enhances cognitive impairment and memory. Furthermore, piracetam increases cerebral blood flow and restores plasma membrane fluidity in the brain allowing neuronal cells to function better [36–41].

Vinpocetine (ethyl apovincaminate) is a synthetic derivative of alkaloid vincamine, extracted from the leaves of the periwinkle plant (*Vinca minor*) and has long been used as a nootropic agent in neurological disorders associated with cerebrovascular diseases. In the brain, vinpocetine acts as a vasodilator by selectively inhibiting Ca2+/calmodulin-dependent cyclic nucleotide phosphodiesterase 1 that enhances cerebral blood flow and cerebral metabolism. In addition, it displays anticonvulsant and neuroprotective effects by blocking voltage-dependent sodium (Na^+^) channels. Furthermore, vinpocetine exhibits a potent anti-inflammatory effect by directly inhibiting IκB kinase complex (IKK) and antioxidant effect by scavenging free radicals [36, 42–44]. Many studies reported on ETP-induced peripheral neuropathy; however, few reports focused on its brain toxicity [24, 45].

This paper investigates the protective potential of taurine, piracetam and vinpocetine in preventing ETP-induced toxicity. The potential of ETP-induced toxicity was investigated by administering multiple doses of ETP intraperitoneally to reach an accumulative dose of 44 mg/kg. Taurine, piracetam and vinpocetine were administered orally to attenuate ETP-induced toxicity. Serum levels of tumour necrosis factor-alpha (TNF-α), interleukin-1 beta (IL-1β), IL-6 and glutathione (GSH) were measured using enzyme-linked immunosorbent assay (ELISA) technique and brain tissues from each group were examined histopathologically using haematoxylin and eosin (H&E) stain and immunohistochemically using glial fibrillary acidic protein (GFAP) antibody stain.

## Materials and methods

### Animals

Thirty female albino rats, 10–12 weeks old and weighing 160–200 grams, were acquired from the experimental animal house at Hawler Medical University, College of Pharmacy (Erbil, Iraq). The animals were housed in polypropylene cages (340 mm × 370 mm) in groups of six, on sawdust in the animal house facility, and kept at a temperature of 25°C ± 3°C, and humidity between 40% and 60% with a 12-hour light and dark cycle. The animals were supplied with rodent chow and had free access to tap water. All rats used in this study were kept in the animal house at the College of Pharmacy/Hawler Medical University and were allowed to adapt to the housing conditions for at least 1 week before starting the experiment. The study was approved by the ethics committee at the College of Pharmacy/Hawler Medical University, with code number 26042022-418-HMU-PH-EC on 22-8-2021, and followed the recommendations of the National Institutes of Health Guide for Care and Use of Laboratory Animals (Publication No. 85-23, revised 1985).

### Drugs and chemicals

TNF-α, IL-1β, IL-6 and GSH ELISA rat kits were purchased from Kiazist Pishro Barman (Iran). ETP vial as etopex 100 mg/5 mL injectable solution manufactured by Deva Pharmaceutics (Istanbul, Turkey), vinpocetine as cavintona 10 mg capsule manufactured by American Medic and Science (Washington, USA), taurine as taurine 500 mg tablet manufactured by Warnke Vitalstoffe GmbH (Wetzlar, Germany) and piracetam as nootropil 800 mg film coated tablet manufactured by Union Chimique Belge (Brussels, Belgium) were purchased from a verified and licensed pharmacy. All drugs in tablet or capsule form were grinded and suspended in distilled water immediately before use. The ETP solution was stored at 4°C and brought to room temperature prior to use. The dose of ETP was selected based on a previous study [45].

### Study design

**Figure 1.**
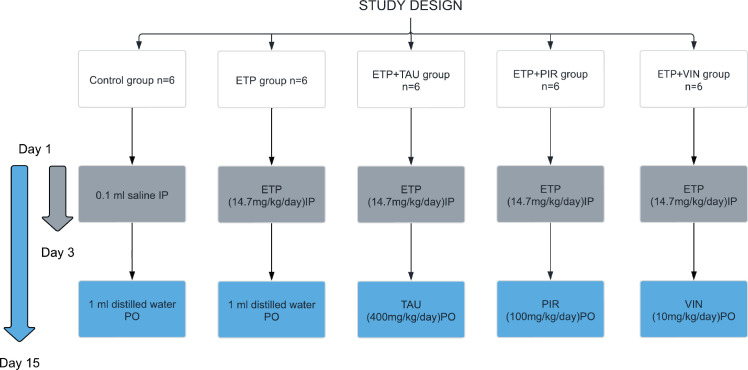
Schematic diagram of experimental groups and treatments in the study.

In this study, the rats were randomly allocated into five groups (Figure 1). The groups were designed as follows: Group I (Control): Received 0.1 mL isotonic saline (0.9% NaCl) intraperitoneally on the 1^st^, 2^nd^ and 3^rd^ day of the experiment and 1 mL of sterile distilled water was given orally via oral gavage daily for 15 days starting from day 1. Group II (ETP): Received ETP (14.7 mg/kg/day) intraperitoneally on the 1^st^, 2^nd^ and 3^rd^ day of the experiment for a total accumulative dose of 44 mg/kg to induce toxicity along with sterile distilled water orally via oral gavage daily for 15 days starting from day 1. Groups III, IV and V served as the treatment groups; they were given ETP as stated in Group II with one of the treatment drugs. Group III (ETP + TAU): Received taurine (400 mg/kg/day) via oral gavage for 15 days starting from day 1 [46]. Group IV (ETP + PIR): Received piracetam (100 mg/kg/day) via oral gavage for 15 days starting from day 1 [36]. Group V (ETP + VIN): received vinpocetine (10 mg/kg/day) via oral gavage for 15 days starting from day 1 [36].

The weights of the rats were recorded on the first day of the experimental study. The rats of each group were observed daily to detect acute signs of ETP-induced toxicity or unusual behaviour/appearance, and mortality. Three hours after the last dose, the rats were weighed and anaesthetised with an intraperitoneal injection of xylazine 10 mg/kg and ketamine 100 mg/kg [47] to render them unconscious which required approximately 2–3 minutes. Blood was withdrawn by using cardiac puncture technique. The brain was harvested, weighed and grossly examined to conduct any abnormalities developed in the organ, and then put in a formaldehyde solution at 37% concentration diluted with 0.9% isotonic saline (1:3) for histopathological and immunohistochemical analysis. The withdrawn blood was placed in gel and clot activator glass tube 5 mL and allowed to incubate at room temperature and clot for 30 minutes. The test tubes were then centrifuged at 3,000 rounds per minutes (RPM) for 10 minutes. This process was repeated if necessary. The obtained serum was placed in Eppendorf tubes, coded with random numbers for a blind investigation, and stored at −20°C for further biochemical analysis.

### Biochemical analysis

The serum levels of GSH were evaluated by ELISA technique using commercial ELISA kit supplied by KiaZIST, Iran and an Elisa Plate Reader (BioTek 800-TS Microplate Reader). The procedure was performed according to Ellman’s method [48]. The test principle comprises a chemical reaction between Ellman’s solution or 5,5’-dithio-bis-(2-nitrobenzoic acid) (DNTB) and reduced thiol groups (-SH) present in GSH to yield nitromercaptobenzoic acid (TNB) that has an intense yellow colour. The intensity of the colour determines the absorbance, measurable at a wavelength of 412 nm, and that is directly proportional to the concentration of GSH in the sample. Thus, the concentration of GSH in the sample was obtained by comparing the absorbance of the sample with a standard curve. Results were expressed as µm/mL.

Serum levels of TNF-α, IL-1β and IL-6 were evaluated by ELISA technique using commercial ELISA kits supplied by KiaZIST, Iran. The procedures were similar for the three cytokines and performed according to the manufacturer’s instructions. First, the standards and serum samples were added to antibody pre-coated plate wells and incubated on a shaker (250 RPM) for 1 hour. Then, the washing solution was diluted with distilled water and the plates were washed three times with washing solution. Incubation and washing were repeated after adding each of the biotin-conjugated antibody (incubated for 1 hour and washed three times) and the horseradish peroxidase (HRP)-Avidin solution (incubated for 30 minutes and washed five times). After that, the substrate was added and incubated for 15 minutes. Finally, the stop solution was added [49]. Absorbance was measured at 450–455 nm, and results were expressed as pg/mL.

### Histopathological analysis

The harvested brains were fixed with formalin for less than 24 hours. Sagittal sections of the brain were embedded in molten paraffin and were serially sectioned at 4 μm thickness using a microtome (Thermo Scientific^TM^ HM E340 Automated Microtome, Germany). The sections were then put on clean slides, deparaffinised and rehydrated using an automated tissue processor (Sakura Histo-Tek^®^ VP1^TM^, Netherlands). The brain sections were mounted on clean slides and stained with H&E. After that, all stained slides were observed under a light microscope (Olympus Light microscope, Germany) for histopathological evaluation and their photomicrographs were captured. All slides were coded with random numbers for a blind investigation by a skillful pathologist. The brain injury was semi-quantitatively graded from (0–4) according to Ibrahim Fouad and Ahmed [27] in which (0) indicated no brain damage, (1) indicated more than 10% brain damage, (2) indicated 20%–30% brain damage, (3) indicated 40%–60% brain damage and (4) indicated more than 60% brain damage.

### Immunohistochemical analysis: GFAP immune expression

GFAP immune expression level in the brain was examined using ready-to-use Dako, Flex Polyclonal Rabbit Anti-GFAP and EnVision FLEX, high pH Link visualizing system supplied by Dako, Denmark. First, the tissue sections were deparaffinised and rehydrated using an automated tissue processor (Sakura Histo-Tek^®^ VP1^TM^, Netherlands). Next, the slides were incubated in a targeted retrieval solution. Then, the slides were incubated in hydrogen peroxidase blocking reagent to minimise antibody binding to nonspecific sites. After that, the slides were incubated with the GFAP antibody, HRP polymer and visualised using 3,3-diaminobenzidine chromogen. Finally, the slides were counter-stained with haematoxylin, dehydrated, cleared and mounted for microscopical examination. All steps of staining and incubation times were preprogrammed and carried out automatically by Dako PT Link Autostainer, Denmark. All slides were coded with random numbers for the blind investigation conducted by a skillful pathologist. The morphological alterations in astrocytes were graded semi-quantitively according to Boos *et al* [50] scoring system illustrated in Table 1. Quantitative analysis of GFAP immunostaining intensity was also determined by measuring the area percent of GFAP-expression of all photomicrographs of brain sections from each group using image analysis software (Image J, version 1.53k, NIH, Bethesda, MD, USA). The images were converted to grayscale (8-bit) and the area percent of GFAP-expression was analysed by changing the contrast to include the brown area of astrocytes and their processes and exclude the background staining from the measurement.

### Statistical analysis

All values were expressed as mean ± standard error of mean (SEM). One-way analysis of variance test followed by least significant difference (LSD) multiple comparison as post hoc test was carried out to compare between means of different groups. Difference between groups was considered statistically significant at *p*-value < 0.05. Statistical analysis of the data was carried out using Statistical Package for the Social Sciences version 22 software.

## Results

### Clinical observation of toxicity signs in different groups

Following administration of drugs, the rats were observed on a daily basis. After administration of the third ETP dose, the rats of the ETP group exhibited decreased physical activity. On days 4 to 15, the rats of the ETP group demonstrated diarrhoea, loss of body weight, red secretions around the eyes and nose, weak response to stimuli, little movement, prostration, low appetite, roughening and thinning and yellowing of the hair coat and hair loss which was commonly seen at the site of injection. In addition to that, the mortality rate in this group was 17% (one rat died out of six). On the other hand, rats of the treatment groups (ETP + TAU, ETP + PIR, ETP + VIN groups) showed decreased physical activity as well as roughening and thinning of the hair but this was less severe than the ETP group while the rats of the control group were healthy and active and did not show any signs of toxicity. Rats of both control and treatment groups did not have any mortalities.

### Taurine, piracetam and vinpocetine mitigated ETP-induced decrease in body weight

At the end of the experiment, there was an increase in the body weight of all rats in all the groups except for the ETP group. The body weight and percentage of body weight changes of ETP administered rats were significantly (*p* < 0.05) reduced by the end of the experiment compared to the rats in the control group. In contrast, taurine, piracetam and vinpocetine significantly (*p* < 0.05) prevented body weight loss in the rats of treatment groups compared to the rats in the ETP group. Moreover, there was no significant (*p* > 0.05) change in the brain weight and relative brain weight percent in rats of all groups (Table 2).

### Taurine, piracetam and vinpocetine mitigated ETP-induced alterations in serum biomarkers

**Figure 2.**
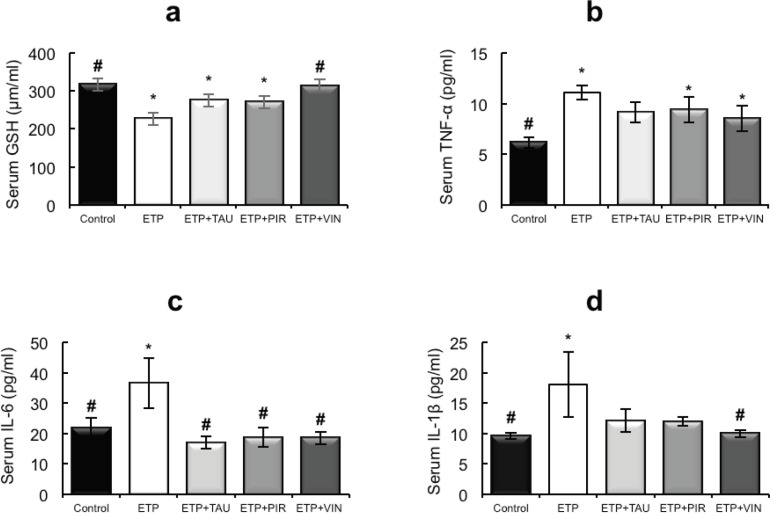
Effect of taurine, piracetam and vinpocetine on the serum levels of (a): GSH, (b): TNF-α, (c): IL-6 and (d): IL-1β of rats treated with ETP. All values are expressed as mean ± SEM. Different signs indicate significances.* Indicates significant difference when compared with control group (*p* < 0.05). # Indicates significant difference when compared with ETP group (*p* < 0.05). Missed signs of significances indicate statistically non-significant groups. ETP, Etoposide; TAU, Taurine; PIR, Piracetam; VIN, Vinpocetine.

Intraperitoneal injection of ETP led to a statistically significant (*p* < 0.05) decrease in the serum levels of GSH relative to the control group. The administration of taurine, piracetam and vinpocetine reversed the depleted serum GSH levels as depicted in Figure 2a. Thus, only vinpocetine was able to significantly (*p* < 0.05) increase serum GSH levels as compared to the ETP-treated group. Figure 2b–d illustrates ETP triggered inflammation by elevation of TNF-α, IL-1β and IL-6 serum levels significantly (*p* < 0.05) in comparison with the control group. Treatment groups reversed the increment in serum TNF-α levels induced by ETP compared with the ETP-treated group but the increment was not significantly (*p* > 0.05) different (Figure 2b). The serum levels of IL-6 in treatment groups reduced significantly (*p* < 0.05) compared to that in ETP group (Figure 2c). The levels of IL-1β were reduced in treatment groups; however, the reduction was statistically significant (*p* < 0.05) by vinpocetine only when compared to ETP group (Figure 2d).

### Effect of taurine, piracetam and vinpocetine on ETP-induced histopathological alterations in brain

**Figure 3.**
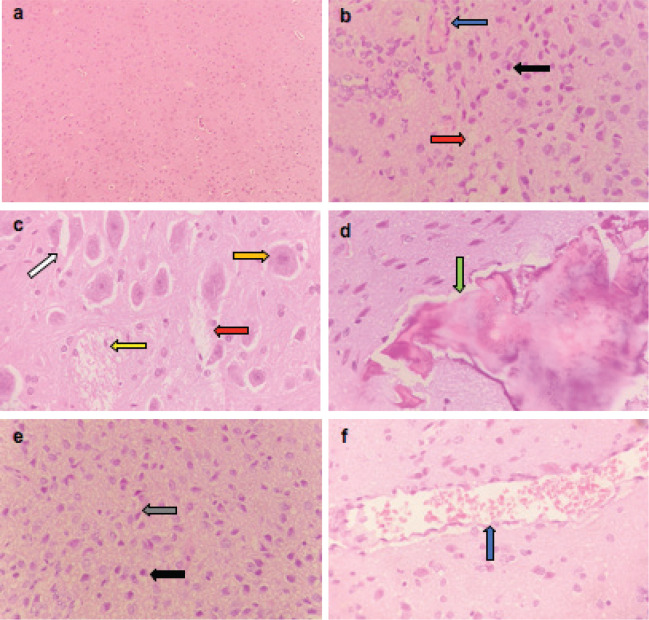
Representative photomicrographs of H&E-stained sections of brain. (a): Control brain is showing normal brain structure. (b–f): ETP-exposed brain is showing (b) vascular congestion (blue arrow), oedema (red arrow) and pyknosis of neurons (black arrow); (c): gemistocytic change (orange arrow), astrocyte proliferation (white arrow), oedema (red arrow) and necrosis of neurons (yellow arrow); (d): bony metaplastic change with calcification (green arrow); (e): gliosis (grey arrow) and pyknosis of neurons (black arrow); (f): vascular congestion (blue arrow). H&E ×400.

**Figure 4.**
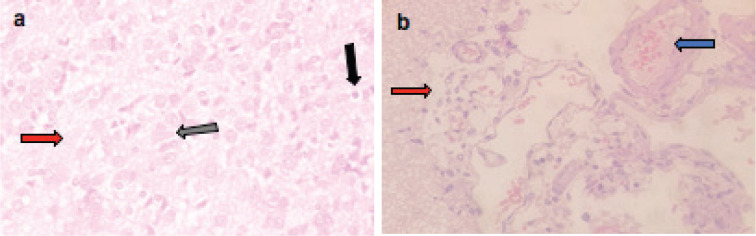
Representative photomicrographs of H&E-stained sections of brain. (a and b): ETP + TAU cotreated brain is showing (a) gliosis (grey arrow), pyknosis (black arrow) of neurons and oedema (red arrow); (b): vascular congestion (blue arrow) and oedema (red arrow). H&E ×400.

**Figure 5.**
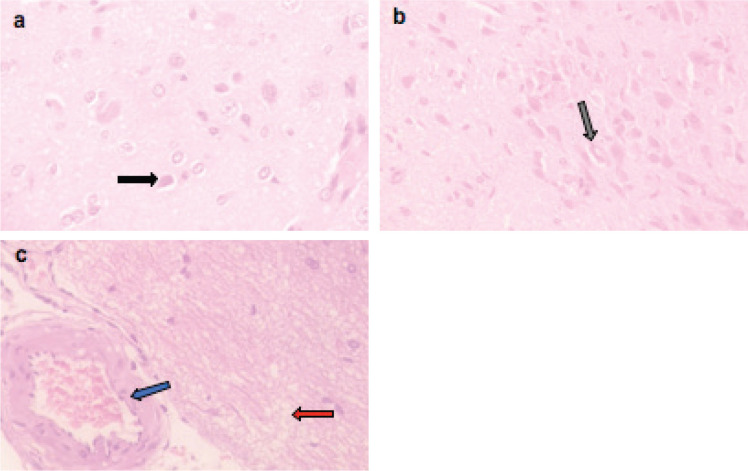
Representative photomicrographs of H&E-stained sections of brain. (a–c): ETP + PIR cotreated brain is showing (a) pyknosis (black arrow) of neurons; (b): gliosis (grey arrow); (c): oedema (red arrow) and vascular congestion (blue arrow). H&E ×400.

**Figure 6.**
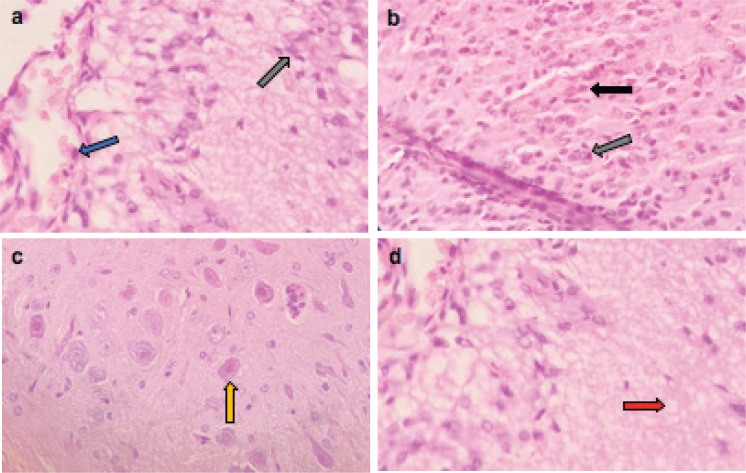
Representative photomicrographs of H&E-stained sections of brain. (a–d): ETP + VIN cotreated brain is showing (a) gliosis (grey arrow) and vascular congestion (blue arrow); (b): pyknosis (black arrow) of neurons and gliosis (grey arrow); (c): gemistocytic change (orange arrow); (d): oedema (red arrow). H&E ×400.

Microscopically, the brains of rats in the control group revealed normal histological structure (Figure 3a). On contrary, the brains of rats in the ETP group exhibited pyknosis and necrosis of neurons, gliosis, gemistocytic change, astrocyte proliferation and bony metaplastic changes with calcification as well as moderate vascular congestion and oedema (Figure 3b–f). Meanwhile, brain sections from ETP-treated rats cotreated with taurine (Figure 4a–b), piracetam (Figure 5a–c) and vinpocetine (Figure 6a–d) alleviated the pathological alterations and showed mild pyknosis of neurons, mild gliosis, vascular congestion and oedema. Table 3 summarises the scores of histopathological alterations in the brains of different groups under study.

### Effect of taurine, piracetam and vinpocetine on ETP-mediated glial cell activation: GFAP-expression in brain

**Figure 7.**
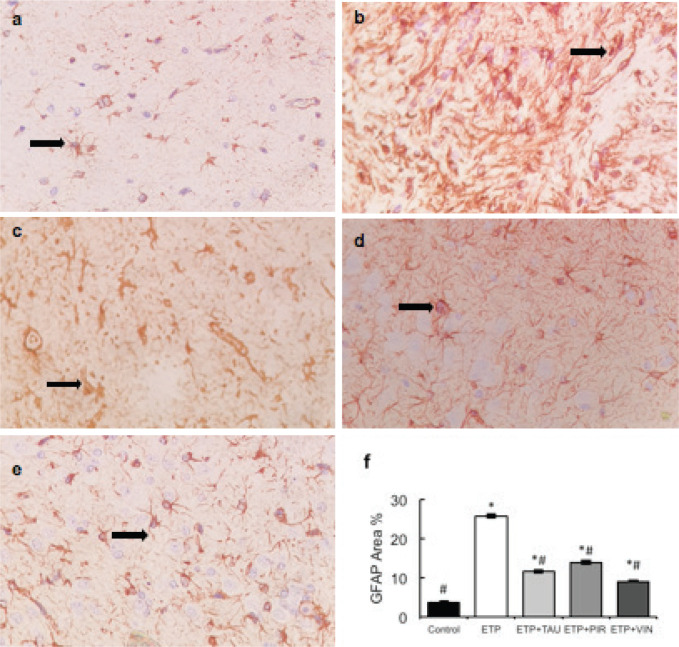
Representative photomicrographs demonstrating the effect of taurine, piracetam and vinpocetine on GFAP immunostaining in ETP-induced brain toxicity in rats. (a): Control brain is showing normal astrocytes and nucleus with light-stained GFAP positive processes (arrow). (b): ETP-exposed brain is showing hypertrophied astrocytes and nucleus with deep-stained GFAP positive processes (arrow). (c): ETP + TAU cotreated brain, (d): ETP + PIR cotreated brain and (e): ETP + VIN cotreated brain all show mild to moderate GFAP-astrocyte expression (arrow). (×400). (f): Area percent of GFAP immunostaining expression in the brain of rats from different groups tested. All values are expressed as mean ± SEM. Different signs indicate significances. * Indicates significant difference when compared with control group (*p* < 0.05). # Indicates significant difference when compared with ETP group. Missed signs of significances indicate statistically non-significant groups. ETP, Etoposide; TAU, Taurine; PIR, Piracetam; VIN, Vinpocetine; GFAP, Glial fibrillary acidic protein.

Microscopically, brain sections of rats in the control group revealed normal astrocytes and nuclei with long, thin and light-stained GFAP positive processes (Figure 7a). While brain sections of ETP-injected rats revealed hypertrophied astrocytes and nuclei with dense, thick, diffused and deep-stained GFAP positive processes (Figure 7b). Furthermore, brain sections from ETP-treated rats cotreated with taurine (Figure 7c), piracetam (Figure 7d) and vinpocetine (Figure 7e) revealed mild to moderate GFAP-astrocyte expression. Table 4 summarises the scores of astrocytic alterations in the brains of different groups being studied. Figure 7f illustrates the area percent of GFAP immunostaining expression. The area percent of GFAP-expression was increased significantly (*p* < 0.05) by 22% in ETP-exposed brains, as compared to the brains of rats in the control group. Co-treatment of ETP-exposed brains with taurine, piracetam and vinpocetine resulted in significant (*p* < 0.05) reduction of area percent of GFAP-expression by 14.2%, 8.9% and 16.8%, respectively, as compared to the brains of rats in ETP treated group.

## Discussion

The immunohistochemical, histopathological and biochemical data from the present study suggest that treatment with taurine, piracetam or vinpocetine mitigates the adverse effects of ETP in experimental rats. The selected treatments alleviated ETP-induced morphological changes and astrogliosis in the brain and mitigated serum levels of GSH, TNF-α, IL-1β and IL-6 involved in ETP toxicity. This study demonstrates the toxic effect of ETP administration on the brain and serum biomarkers. In addition, it exhibits the efficacy of taurine, piracetam and vinpocetine in preventing ETP toxicity in female albino rats.

The lethal dose 50 for intravenous administration of ETP in rats is evidenced to be 68 ± 3.4 mg/kg [21]. In the present study, a total cumulative dose of 44 mg/kg injected intraperitoneally was proved to induce brain injury in rats. This dose was selected based on an earlier study where it was concluded that a single intravenous injection of 44 mg/kg of ETP-induced sensory neuropathy in laboratory animals [45].

In the present investigation, ETP triggered a significant decrease in body weight in rats compared to the control group. An earlier study has also reported a loss in body weight in mice due to an intraperitoneal injection of ETP [51]. This decrease in body weight could strongly be related to the reduced food intake and occurrence of diarrhoea in the rats treated with ETP alone. The administration of each of taurine, piracetam and vinpocetine with ETP was found to improve food intake and consequently prevent weight loss in treatment groups.

Enhanced production of proinflammatory cytokines has been involved in neurotoxicity. Among the proinflammatory cytokines, TNF-α, IL-1β and IL-6 are extremely potent and considered to be the major cytokines responsible for inflammatory reactions [52]. Thus, in the present work, the serum levels of TNF-α, IL-1β and IL-6 were examined. Results depicted that ETP significantly increased serum levels of TNF-α, IL-1β and IL-6 compared to the control group. These findings are in harmoniousness with the earlier investigations which demonstrated that treatment with doxorubicin or cisplatin leads to peripheral elevation of TNF-α, IL-1β and IL-6 [28, 52]. Studies proved that increased plasma TNF-α is the most critical link between peripheral and CNS damage following doxorubicin treatment. TNF-α can cross the BBB leading to microglial activation, increased reactive oxygen species (ROS) and further TNF-α release in the brain, resulting in mitochondrial dysfunction, neuroinflammation and neuronal apoptosis [28, 53]. Therefore, suppression of serum TNF-α is expected to aid in the prevention of brain damage. This is supported by our findings that elevated serum levels of TNF-α by ETP were reduced upon treatment with either taurine, piracetam or vinpocetine. The current work results showed a more potent anti-inflammatory effect of vinpocetine by significantly reducing IL-1β and IL-6 serum levels.

It has been proven that the metabolites of ETP mainly ortho-quinone as well as short-lived intermediates (phenoxy and semiquinone free radicals) are involved in DNA damage and oxidative injury. These free radicals react with intracellular reducing agents mainly thiol, thus, modulating the toxicity of the drug. This interaction prevents further transformation of ETP to its original form and causes accumulation of ROS which directly causes oxidative stress in peripheral tissues and may subsequently lead to neurotoxicity [1, 10, 54]. In addition, the overproduction of ROS has been found to further accelerate the release of proinflammatory cytokines [52]. Therefore, oxidative stress and inflammation may have interrelated mechanisms in the development of ETP toxicity. GSH is the main thiol present in high concentrations in all mammalian cells and represents a defence mechanism against injury caused by ROS. It has been proven that GSH forms a conjugate with ETP-quinone and scavenges possibly formed radical species [1, 54, 55]. Based on that, the relationship between serum levels of GSH and ETP toxicity was examined. Results demonstrated a significant reduction in serum levels of GSH in ETP-treated rats compared to the control group. This finding agreed with an earlier report on the level of GSH in brain tissue of ETP-induced toxicity [1]. Treatment groups were able to restore the decreased levels of GSH. The ability of taurine, piracetam and vinpocetine to ameliorate the rise in ROS and the reduction in antioxidant levels was reported in several previous animal studies [33, 36, 39, 40, 42, 56]. However, the current result showed that vinpocetine is a better free radical scavenger than piracetam and taurine. Several factors may have been responsible for the antioxidant and anti-inflammatory effects of the drugs in this study, including the selected doses, route of administration and the period of the treatment.

The degree of ETP-induced brain injury was estimated by examining GFAP immunohistochemically. GFAP is a marker protein of astrocytes, the most abundant subtype of glial cell in the brain. GFAP is an intermediate filament protein responsible for maintaining the morphology and function of mature astrocytes. It is overexpressed during gliosis associated with brain injury and diseases [57]. Astrocytes respond to neuroinflammation and brain injury through a process called astrogliosis characterised by astrocyte proliferation and cell body hypertrophy. Moreover, upregulation of specific structural proteins, mainly GFAP depends on the severity of the injury [58]. Herein, GFAP expression was increased in the ETP-exposed brains, as compared to the control group. Another anticancer agent that induces damage to astrocytes and GFAP alteration in laboratory animals is doxorubicin [27, 53, 59]. Consistently, with the immunohistochemical results, the histological findings confirmed the ability of ETP to induce brain injury. The histopathological examination of ETP-neurotoxicated brains showed pyknosis and necrosis of neurons, gliosis, gemistocytic changes, astrocyte proliferation and bony metaplastic changes in brain with calcification as well as moderate vascular congestion and oedema. In accordance with the biochemical findings, taurine, piracetam and vinpocetine administration to ETP treated rats decreased GFAP immune expression and exhibited improvement of the histologic appearance of the brain tissue, as compared to the rats in the ETP treated group; confirming the protective effect of these drugs against ETP-induced brain injury.

## Conclusion

Results of the present study concluded that ETP induced brain damage evidenced by immunohistochemical and histological alterations. In addition to that, ETP enhanced the production of serum proinflammatory cytokines (TNF-α, IL-1β and IL-6) and depleted serum levels of GSH. Co-treatment with taurine, piracetam or vinpocetine provided effective protection against brain injury and toxicity induced by ETP. They alleviated pathological alterations and GFAP immune expression in the brain. Furthermore, they downregulated the overproduced serum proinflammatory cytokines and restored serum GSH level, confirming their anti-inflammatory and antioxidant actions. According to our results, vinpocetine appears to be more potent than taurine and piracetam in ameliorating brain injury and alterations in serum biomarkers induced by ETP. Further studies need to validate the effect of taurine, piracetam or vinpocetine on proinflammatory cytokines and more oxidative stress biomarkers levels in brain. Moreover, studies are required to cover the neurophysiological, neurochemical and behavioural changes that may result from ETP and whether co-administration of taurine, piracetam or vinpocetine mitigates these toxicities without compromising on ETP anticancer activity.

## Conflicts of interest

The authors declare that there are no conflicts of interest.

## Funding

Hawler Medical University/Animal House provided the facility to conduct the animal studies. Researchers were self-funded to carry out the rest of the experimental procedures.

## Figures and Tables

**Table 1.** Semi-quantitative scoring system for astrocytic alterations in the central nervous system (CNS) according to GFAP staining [50].

Morphologic parameters	The severity of astrocytic alterations			
	Non-altered	Mild	Moderate	Severe
Apparent cellular alteration	No	No or discrete	Moderate	Yes
Nucleus alteration	No	Mild increase in volume	Moderate increase in volume	Severe increase in volume, binucleated cells (gemistocytes)
Cytoplasm	Few mildly stained cells	Mildly stained	Moderately stained	Severely stained
Processes	Long, thin, well-ramified	Long, thin, well-ramified	Long, moderately thickened	Thickened, trespassing other cells processes, glial scar formation
Grades	0	1	2	3

CNS, Central nervous system; GFAP, Glial fibrillary acidic protein

**Table 2.** Effect of taurine, piracetam and vinpocetine on changes in body and brain weight of rats treated with ETP.

Group	Control	ETP	ETP + TAU	ETP + PIR	ETP + VIN
Initial body weight (g)	184.5 ± 6	193.8 ± 6.25	172.8 ± 3.4	186.8 ± 6	172 ± 4.7
Final body weight (g)	192 ± 5	180.8 ± 7	187 ± 3.4	199.8 ± 6.48	183 ± 4.5
Body weight change (g)	7.5 ± 2.2^b^	−13 ± 1.2^a^	14.25 ± 2.3^b^	13 ± 3^b^	11 ± 4.4^b^
Body weight change (%)	4.1 ± 1.3^b^	−6.8 ± 0.8^a^	8.3 ± 1.4^b^	7 ± 1.7^b^	6.5 ± 2.7^b^
Brain weight	2.8 ± 0.04	2.9 ± 0.05	2.9 ± 0.01	2.9 ± 0.04	2.8 ± 0.05
Relative brain weight (%)	1.5 ± 0.04	1.6 ± 0.08	1.6 ± 0.05	1.4 ± 0.06	1.6 ± 0.05

All values are expressed as mean ± SEM

Different signs indicate significances

a *p* < 0.05 significantly different when compared with control group

b *p* < 0.05 significantly different when compared with ETP group

Missed signs of significances indicate statistically non-significant groups

ETP, Etoposide; TAU, Taurine; PIR, Piracetam; VIN, Vinpocetine

**Table 3.** Semi-quantitative scoring of histopathological alterations in the brains of different groups.

Histopathological alterations in the brain	Experimental groups				
	Control	ETP	ETP + TAU	ETP + PIR	ETP + VIN
Necrosis of neurons	0	2	0	0	0
Pyknosis of neurons	0	2	1	1	1
Gliosis	0	2	1	1	1
Gemistocytic change	0	2	0	0	1
Astrocyte proliferation	0	2	0	0	0
Bony metaplastic change with calcification	0	2	0	0	0
Oedema and vascular congestion	0	2	1	1	1

ETP, Etoposide; TAU, Taurine; PIR, Piracetam; VIN, Vinpocetine

**Table 4.** Semi-quantitative scoring of astrocytic alterations in the brains of different groups under study.

Experimental groups	Control	ETP	ETP + TAU	ETP + PIR	ETP + VIN
Degree of astrocytic alteration	1	3	2	2	2

ETP, Etoposide; TAU, Taurine; PIR, Piracetam; VIN, Vinpocetine
